# Ulnar nerve anteposition with adipofascial flap, an alternative treatment for severe cubital syndrome

**DOI:** 10.1186/s12893-023-02173-6

**Published:** 2023-09-04

**Authors:** Michele Riccio, Pasquale Gravina, Pier Paolo Pangrazi, Valentina Cecconato, Antonio Gigante, Francesco De Francesco

**Affiliations:** 1 Department of Reconstructive Surgery and Hand Surgery, University Hospital (AOU Ospedali Riuniti delle Marche), Via Conca 71, Torrette Di Ancona, Ancona, 60123 Italy; 2 Clinical Orthopedics, Department of Clinical and Molecular Science, School of Medicine, Università Politecnica Delle Marche, Via Tronto, 10/a, 60126 Ancona, AN Italy

**Keywords:** Ulnar nerve, Cubital syndrome, Adipofascial flap, Surgical techniques

## Abstract

**Background:**

Ulnar nerve entrapment at the elbow is the second most common cause of nerve entrapment in the upper limb. Surgical techniques mainly include simple decompression, decompression with anterior transposition and medial epicondylectomy.

**Methods:**

We performed decompression with anterior transposition and protected ulnar nerve by adipofascial flap (a random flap with radial based vascularization, harvested through the avascular plane of Scarpa’s fascia. We analyzed patients who underwent ulnar nerve ante-position from 2015 to 2022 according to inclusion and exclusion criteria for a total of 57 patients. All patients included were graded on the McGowan's classification Messina criteria and the British Medical Research Council modified by Mackinnon and Dellon.

**Results:**

The average McGowan’s score was 2.4 (± 0.6), Messina’s criteria 91.2% indicated a satisfactory or excellent result, sensibility at 6 months was 98.5% S3 or more. A preferential technique has not yet been defined.

**Conclusions:**

The adipofascial flap offers numerous advantages in providing a pliable, vascular fat envelope, which mimics the natural fatty environment of peripheral nerves and creates favorable micro-environmental conditions to contribute to neural regeneration via axon outgrowth.

## Background

The ulnar nerve is a motor and sensitive nerve that originates from C8-T1 nerve roots, which can be subject to compression in several points, resulting in clinical signs that may vary in relation to the specific compression area. From C8-T1 nerve roots converge to form the medial cord of the brachial plexus [1–4] with the first point of compression at the arcade of Struthers, a thick septum that links the medial intermuscular septum to the triceps medial head [3], subsequently passing through the cubital tunnel where further compression may occur in the Osborne’s Ligament, a ligament spanning from the olecranon and medial epicondyle progressing with the fascia that connects the two heads of the flexor carpi ulnaris (FUC) [5]. The FUC has two fascia, a superficial fascia and a deep fascia, the former is known as the Osborne fascia and the latter as the Amadio and Beckenbaugh fascia [6], both are points of nerve compression; compression can also arise at the Guyon’s canal. The most common site of compression is the elbow, giving rise to the “Cubital Syndrome” [7, 8].

Clinical presentation of cubital syndrome involve 4^th^ and 5^th^ fingers paresthesia, muscular hand weakness, muscular atrophy and claw deformity [3]. Clinical signs observable are Froment sign [3],Wartenberg sign [9]. Tinel sign can be positive at the elbow [10, 11]. Clinical Diagnosis is confirmed by strumental exams. XR may be assessed in post-traumatic patients [10, 12]. A cervical spine MRI is required to exclude cervical radiculopathy when suspect occurs; more important are Ultrasonography (US) for morphological nerve assessment [13] and electromyography (EMG) to confirm the site of compression and to investigate the degree of ulnar damage [3]. Treatment may be conservative or surgical. Early stage of conservative treatment involves behavior modifications, non-steroidal anti-inflammatory drugs (NSAIDs), night splints, physical therapy, and corticosteroid injection [4]. Surgical treatment is performed in case of conservative treatment failure with different technique proposals such as simple decompression, decompression with anterior transposition (submuscular, intramuscular, or subcutaneous) and medial epicondilectomy [14]. Simple decompression consists in a release of all compression structures along the nerve course at the elbow maintaining its normal position posterior to the medial epicondyle. Subcutaneous transposition implies creating a subcutaneous pathway of the nerve anterior to the medial epicondyle and over the muscular belly, can be performed with [15] or without the add of adipo-fascial flap. Anterior intramuscular transposition includes elevation of the flexor/pronator from the origin at the epicondyle and transposition of the nerve under all muscular mass; medial epicondylectomy is an in situ decompressive technique involving the removal of the medial epicondyle prominence subperiosteally to release pressure on the nerve [16]. A preferential technique has not been defined [17] but herein we propose the procedure we commonly use in advanced ulnar nerve nephropathy at the elbow which represents the evolution of subcutaneous transposition, accrual to an adipofascial flap to protect and feed the nerve as well as to determine an improved gliding surface from the skin with no involvement of the ante-posed nerve.

## Materials and methods

### Clinical study

**Fig. 1:**
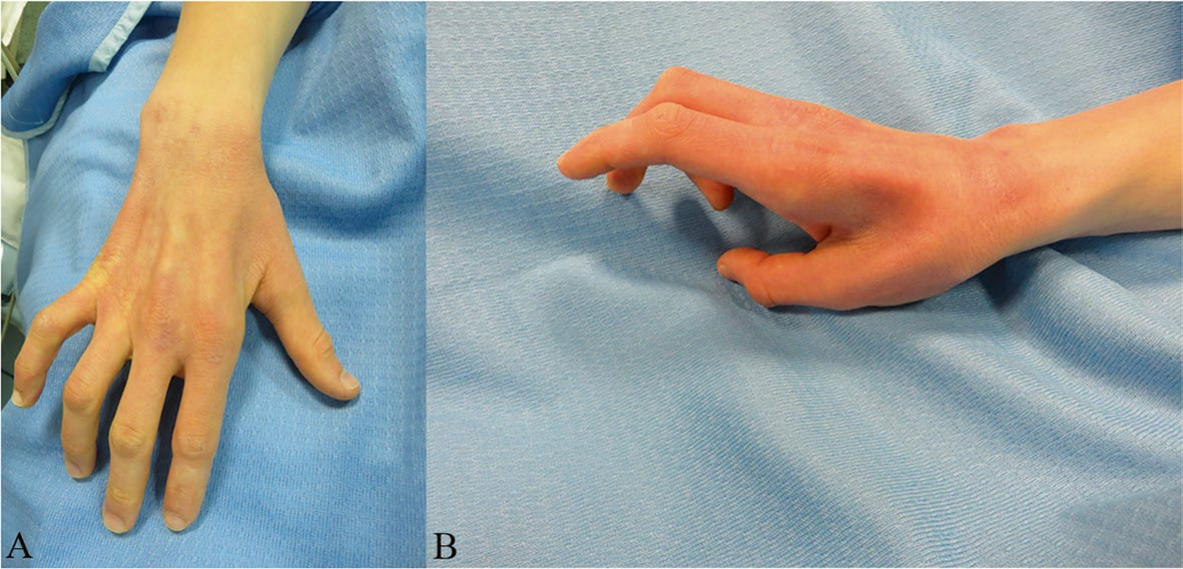
Clinical presentation of ulnar nerve neuropathy McGowan III; **A** shows “squared hand”, Wartenberg sign, interosseus atrophy; **B** shows detail on 1° web space athrophy

In this retrospective study, we analyzed all patients who underwent ulnar nerve ante-position from 2015 to 2022 according to the following selection criteria: inclusion criteria comprised men and women with ulnar nerve entrapment at the elbow confirmed by electromyography with clinical signs for at least 6 months subsequent to unsuccessful conservative treatment which included a follow- up period of 12 months. Exclusion criteria defined patients affected by polyneuropathy, diabetes, previous surgery at the elbow, previous elbow fracture, post-traumatic ulnar nerve lesion at the elbow. None of these patients possessed remarkable medical histories. The clinical outcomes varied from sensibility defect to muscular atrophy. Clinical presentation of advanced ulnar neuropathy of the hand precedent to surgical procedures is shown in Fig. 1.

### Study population

In this study we analyzed 66 patients, who had undergone surgery from 2015 to 2022 in our hospital, 9 of these patients were lost to follow- up. Consequently, the study population was comprised of 57 patients with a mean age of 58, including 21 females, 36 men, three bilateral cases and with mean symptom duration of 24 months and mean follow- up of 12 months (Table 1).

**Table 1:** Study population

NUMBER	AGE	MEN	FEMALE	MEAN SYMPTOMS DURATION (Months)	MEAN FOLLOW- UP (Months)	BILATERAL CASE	HAND DOMINANCE
57	58 (range 42–86)	37	20	24 ± 6	12	3	35 dominant 19 non dominant 3 bilateral

### Surgical technique

**Fig. 2:**
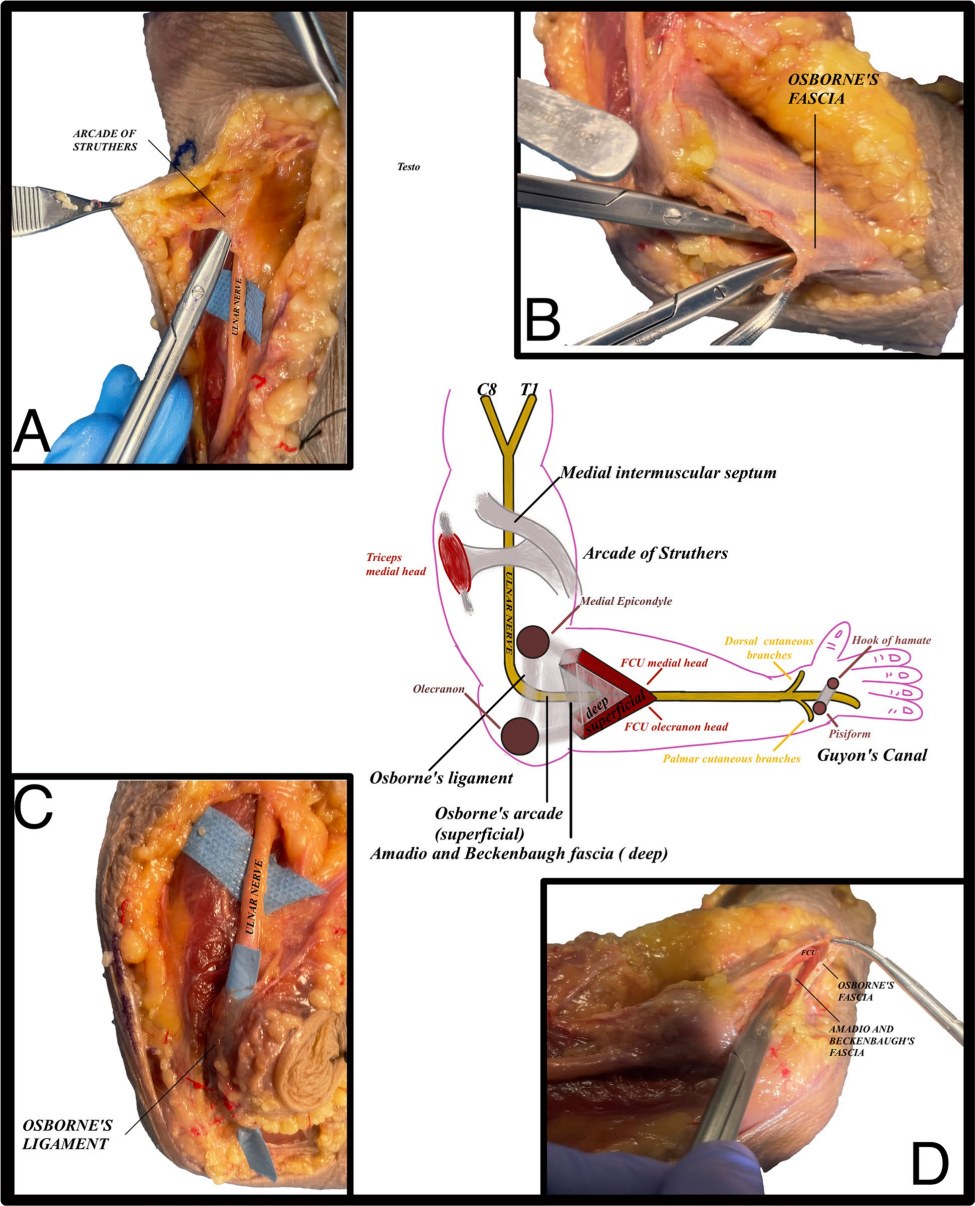
**A** shows a schematic representation of ulnar nerve entrapment at the elbow, and in correspondence with a cadaveric model; **B** Arcade of Struthers; **C** Osborne’s ligament, Osborne’s arcade; **D** Amadio and Beckenbaugh fascia

Main points of compression to be released for ulnar nerve entrapment at the elbow are Struther’s arcade, Osborne’s ligament, Osborn Fascia and Amadio and Beckenbaug fascia (Fig. 2).

**Fig. 3:**
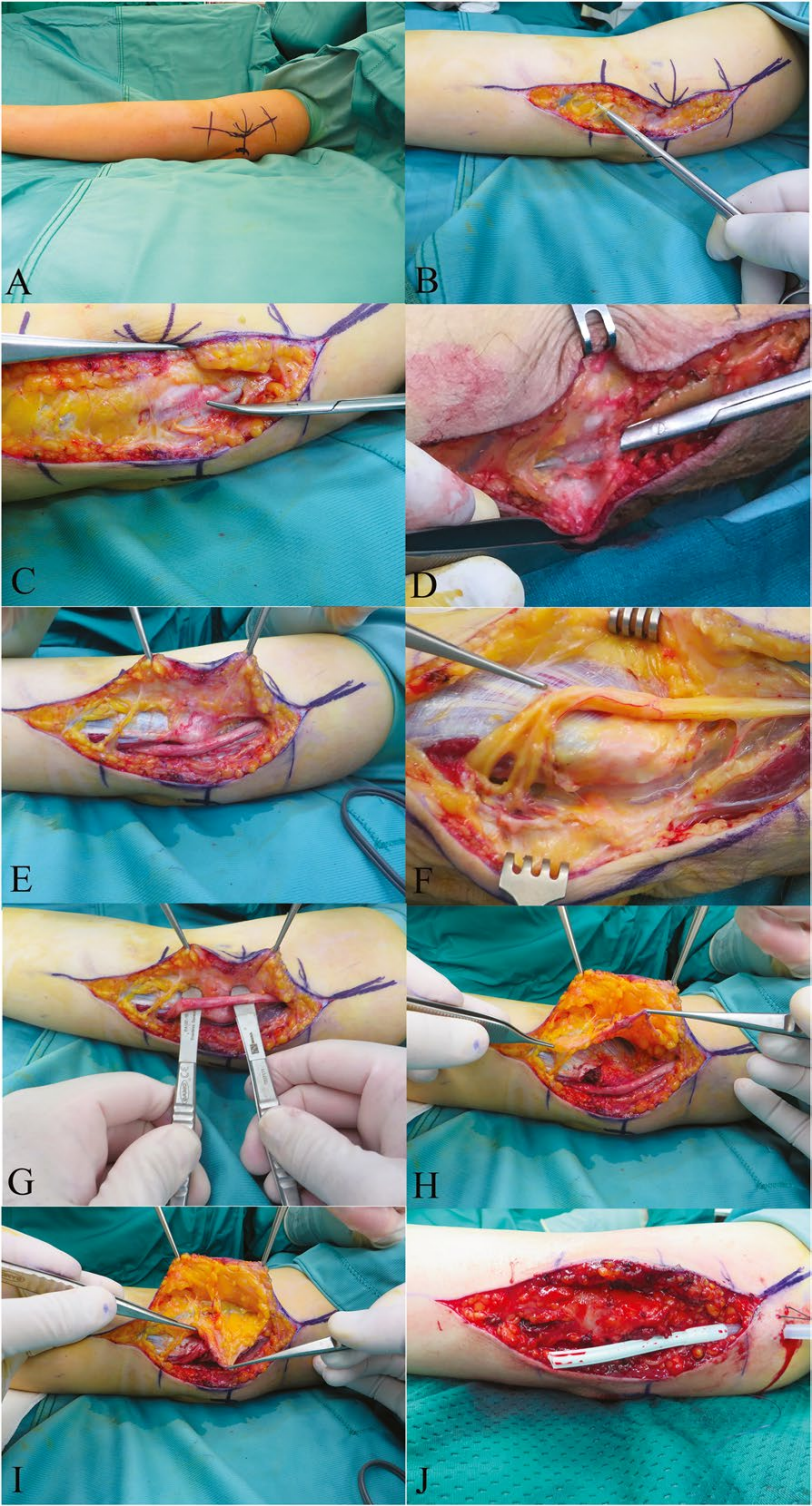
Surgical tecnique. **A** incision of 10–12 cm on the medial aspect of the elbow; **B** identification of sensitive brunch of medial cutaneous nerve; **C** identification of deep fascia; **D** Osborne lIment; **E** ulnar nerve free; **F** motor brunch for flexor ulnaris carpis; **G** free nerve with no kinking after ante position; **H** muscular belly bed prepared and adipofascial flap harvested; **I** ulnar nerve covered with the flap; **J** flap vascularized and drainage positioned

The in vivo procedure begins with an incision of 10–12 cm, in ischemia of the upper limb, on the medial aspect of the elbow, in a region where the scar does not interfere with the normal elbow stand (Fig. 3A), care must be taken in identifying the sensitive brunch of the medial cutaneous nerve (Fig. 3B), the deep fascia is identified (Fig. 3C), the Osborne ligament is identified (Fig. 3D) and sectioned, so that the ulnar nerve is freed together with the Osborne’s fascia and Amadio and Beckenbough fascia distally, and Struther’s arcade distally (Fig. 3E). Caution must be exercised to prevent damage of the motor brunch for flexor ulnaris carpis (Fig. 3F), the ulnar nerve is now free and can be mobilized, the ulnar nerve is evaluated for undesirable kinking after ante position, (Fig. 3G), a single incision in the muscular belly of the epitrochlearis muscle is performed to create a vascularized bed for the nerve and the random adipofascial flap is harvested through the avascular plane of Scarpa’s fascia (Fig. 3H) (the Scarpa’s fascia is the subcutaneous fascia, allowing for an adequate deep venous system in the flap), the ulnar nerve is now covered and the flap attached to the epitrochlear structures to avoid regression of the nerve to original groove (Fig. 3I). The tourniquet is removed, the well- vascularized flap from the anterior aspect of the elbow is attached to the epicondyle using an absorbable stitch, appropriate hemostasis is performed and a drainage is left to avoid postoperative hematoma and to reduce adherences (Fig. 3J). The final procedure involves two different layers spearing the nerve from the flap, and the flap from the skin. The drainage is left for approximately one day, or until the reservoir is filled with less than 30 cc of blood in one day to avoid post-operative hematoma. The patient’s elbow is then freed and stitches are removed at 15 days after surgery. Casts or similar medical appliances are not required.

### Assessment: Mcgowan’s scale and other scales

All patients included in the study were graded according to the McGowan's classification (Table 2) for preoperative assessment, considering a 3-stage classification which provides objective data concerning sensitivity of the ulnar sensory innervation and ulnar innervated intrinsic muscle strength. It considers, moreover, stage I with a minimally affected lesion by sensory defect only and absence of motor weakness in the hand, stage III determines a severe lesion with paralysis of ulnar intrinsic muscles, stage II is indicative of an intermediate lesion [18]. For postoperative assessment we used the Messina’s criteria (Table 3), a 4-stage score which considers sensitive and motor symptoms ranging from “poor” in case of symptoms yielding no improvement or worsening, “fair” for improvement but with residual motor loss, “good” in case of occasional tenderness to the incision site and “excellent” for complete resolution [19]. We considered objective data during follow-up according to the British Medical Research Council, and modified by Mackinnon and Dellon (Table 4) which implies only sensitive data with a 7- stage score ranging from no recovery of sensitivity in S0 to complete recovery in S4 [20]. Other data included recurrence, postoperative hematoma, and scar sensitivity at 6 months. Data are displayed in Table 5.

**Table 2:** McGowan’s classification for preoperative assessment

McGowan's classification (PRE-OPERATIVE)	
0	No Symptoms
I	Minimal lesions, with no detectable motor weakness of the hand
II	Intermediate lesions
III	Severe lesions, with paralysis of one or more of the ulnar intrinsic muscles

**Table 3:** Messina's criteria for postoperative assessment

Messina's criteria ( POST OPERATIVE)	
Excellent	Complete resolution of symptoms with no postoperative motor or sensory deficit
Good	General resolution of symptoms but occasional tenderness at the incision site
Fair	Improvement after surgery but with persistent sensory changes, residual motor loss, muscle wasting, or persistent claw deformity
Poor	No improvement after the surgical procedure or worsening of symptoms

**Table 4:** British Medical Research Council (BMRC), as modified by Mackinnon and Dellon for sensory grading

GRADE	RECOVERY OF SENSIBILITY	s2PD	m2PD
S0	No recovery of sensibility in the autonomous zone of the nerve		
S1 +	Recovery of deep cutaneous pain sensibility within the autonomous zone of the nerve		
S2	Recovery of superficial pain and some touch sensibility		
S3	As S2 but with overresponse	> 15	> 7
S3 +	As S3 but localization of the stimulus is good with imperfect recovery of 2PD	7–15	4–7
S4	Complete Recovery	2–6	2–3

**Table 5:** Data include age, McGowan’s and Messina’s score, postoperative hematoma, BMRC, recurrence, postoperative hematoma

PATIENT	AGE	McGOWAN'S	MESSINA'S	POSTOPERATIVE HEMATOMA	BMRC	RECURRENCE	SENSITIVE SCAR T180
1	45	1	EXCELLENT	NO	s4	NO	NO
2	47	2	EXCELLENT	NO	S4	NO	NO
3	72	3	GOOD	NO	s3 +	NO	NO
4	45	2	EXCELLENT	NO	S4	NO	NO
5	74	3	GOOD	NO	S3 +	NO	NO
6	54	3	GOOD	NO	S3	NO	NO
7	77	2	EXCELLENT	NO	S4	NO	NO
8	57	2	EXCELLENT	NO	S4	NO	NO
9	73	3	GOOD	NO	S3 +	NO	NO
10	78	2	EXCELLENT	NO	S3 +	NO	NO
11	59	3	EXCELLENT	NO	S4	NO	NO
12	84	2	EXCELLENT	NO	S3 +	NO	NO
13	49	2	EXCELLENT	NO	S3 +	NO	NO
14	45	2	EXCELLENT	NO	S3 +	NO	NO
15	61	3	GOOD	NO	S3 +	NO	NO
16	86	3	GOOD	NO	S3 +	NO	YES
17	76	3	GOOD	SI	S3	NO	NO
18	72	2	GOOD	NO	S3 +	NO	NO
19	65	1	GOOD	SI	S3 +	NO	NO
20	47	2	EXCELLENT	NO	S4	NO	NO
21	51	2	EXCELLENT	NO	S4	NO	NO
22	59	1	GOOD	NO	S4	NO	NO
23	45	3	GOOD	NO	S2	NO	NO
24	54	2	EXCELLENT	NO	S3 +	NO	YES
25	49	2	EXCELLENT	NO	S4	NO	NO
26	46	2	GOOD	SI	s3 +	NO	NO
27	45	2	GOOD	NO	S3 +	NO	NO
28	42	1	EXCELLENT	NO	S4	NO	NO
29	57	2	GOOD	NO	S3 +	NO	NO
30	49	2	EXCELLENT	NO	S4	NO	NO
31	49	3	EXCELLENT	NO	S3 +	NO	NO
32	51	3	EXCELLENT	NO	S4	NO	NO
33	68	3	FAIR	NO	S3	NO	YES
34	48	3	EXCELLENT	NO	S3 +	NO	NO
35	61	2	EXCELLENT	NO	s3 +	NO	NO
36	58	2	GOOD	NO	S3 +	NO	NO
37	74	3	FAIR	NO	s3 +	NO	YES
38	51	2	GOOD	NO	S3 +	NO	NO
39	80	3	GOOD	NO	S3 +	NO	NO
40	76	3	EXCELLENT	NO	S3 +	NO	NO
41	57	1	GOOD	NO	S4	NO	NO
42	53	3	EXCELLENT	NO	S3 +	NO	NO
43	73	3	EXCELLENT	NO	S4	NO	NO
44	48	3	GOOD	NO	S3 +	NO	NO
45	49	3	FAIR	NO	S3	NO	YES
46	51	3	GOOD	NO	S3 +	NO	NO
47	52	2	GOOD	NO	s3 +	NO	NO
48	78	3	FAIR	NO	S3	NO	NO
49	52	2	EXCELLENT	NO	S3	NO	NO
50	54	3	GOOD	NO	S4	NO	NO
51	49	2	EXCELLENT	NO	S3 +	NO	NO
52	52	2	EXCELLENT	NO	S3 +	NO	NO
53	51	3	GOOD	NO	S4	NO	NO
54	57	3	GOOD	NO	S3 +	NO	NO
55	44	2	GOOD	NO	S3 +	NO	NO
56	73	3	FAIR	NO	S3	NO	YES
57	50	3	EXCELLENT	NO	S3 +	NO	NO

### Search strategy

**Fig. 4:**
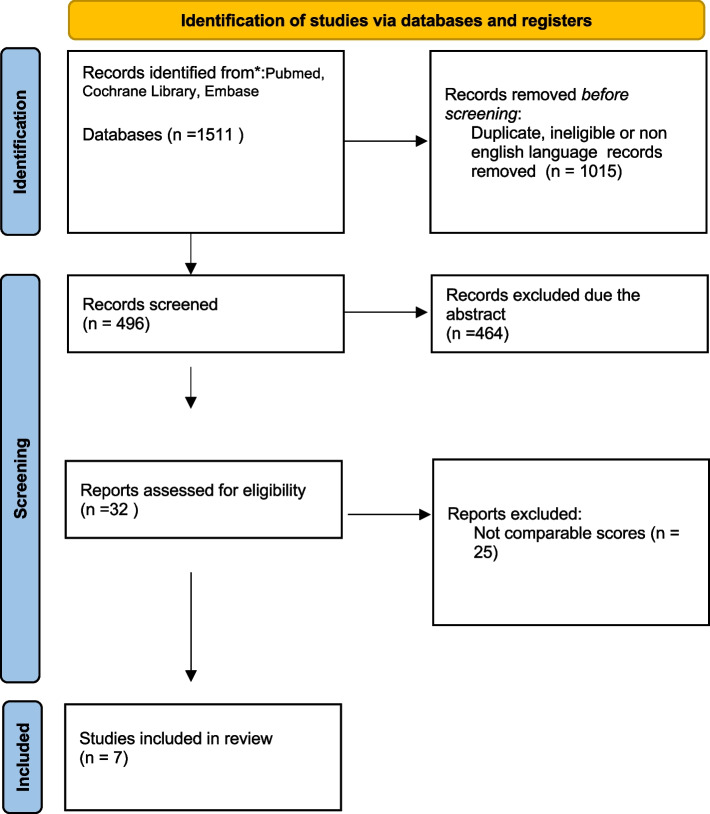
The picture shows the prisma flow diagram used for searching process

(i) Search site: Articles are from PubMed, a database of papers on biomedical science. (ii) Database: MEDLINE(Pubmed), the Cochrane Library, EMBASE and Scopus. (iii) Ulnar nerve entrapment, cubital syndrome (iv) Boolean algorithm: (“cubital syndrome” OR “ulnar nerve entrapment”) AND (“surgical techniques” OR “decompression” OR “decompression with transposition” OR “medial epicondylectomy”) AND (“Adipofascial flap”). (v) Retrieval timeframe: We searched the selected journals published after 1995. (vi)Inclusion and exclusion criteria: The search process was performed as PRISMA flow diagram (Fig. 4).

### Data extraction and analysis

Two observers (P.G. and F.D.F.) independently searched and collected data from the included studies. Any discordances were solved by consensus with a third author (M.R.). All data concerning surgical techniques of ulnar nerve entrapment (cubital syndrome) were carefully reviewed and collected. We compared our data with 25 years of literature excluding studies before 1995. According to a meta-analysis published in the Journal of Hand and Microsurgery in 2019 [21], different evaluation methods on behalf of hand surgeons are available regarding ulnar nerve preoperative and postoperative developments. In this meta-analysis involving 1511 studies, 17 cases were analyzed for comparison of the ulnar nerve in situ decompression and anterior transposition, among which 7 studies involved data using scores comparable with our investigations [22–28] (Table 6). We thus conducted a comparative analysis of preoperative (Mc Gowan 0-I-II-III) and postoperative outcomes, using an assessment of “improvement” or “lack of improvement”. Numerous studies have analyzed results in terms of “poor-fair-good–excellent”, we redefined “good–excellent” as “improvement” and “poor-fair” as “lack of improvement”.

**Table 6:** Study characteristics

STUDY	YEAR	JOURNAL	DESIGN	N	GENDER(%Male)	MEAN AGE	AVARAGE FOLLOW UP (MONTHS)	OUTCOME MEASURE
**Kamat et al. **[28]	2014	*Acta Neurochirur*	Retrospective review	480	47	50	3	McGowan score
**Bacle et al. **[22]	2014	*Ortho Traumatol Surg Res*	Retrospective review	409	44	56.3	92	Patient satisfaction
**Sousa et al. **[26]	2014	*Rev Bras Ortop*	Retrospective review	97	60.3	52.2	10.3	Wilson and Kout score
**Mitsionis et al. **[25]	2010	*J Shoulder Elbow Surg*	Retrospective review	73	N/A	51	37	McGowan score
**Biggs and Curtis** [23]	2006	*Neurosurgery*	Prospective randomized	44	23	58.9	42	McGowan score
**Taha et al. **[27]	2004	*Neurosurgery*	Retrospective review	38	21	63	48	Gabel score
**Bimmler and Meyer** [24]	1996	*Ann Chir Main Memb Super*	Retrospective review	79	31	45	76	McGowan score

*Abbreviations*: *N/A* not applicable

### Statistical analysis

The Kolmogorov–Smirnov test revealed non-normally distributed data; therefore, all statistical analyses were carried out according to a non-parametric approach. Test of proportions for categorical variables between the two groups (chi square) and test of means using non-parametric (Wilcoxon rank sum test) statistical procedures were used. The threshold for statistical significance was set at *p* < 0.05. Repeatability is represented as a standard deviation to calculate the differences between measurements using SPSS version 16.0 software (SPSS Inc., Chicago, IL, USA).

## Results

According to the requirements of this retrospective paper, seven papers have benn selected, analyzed and compared with our techniques.

### Assessment: Mcgowan’s scale and other scales

**Fig. 5:**
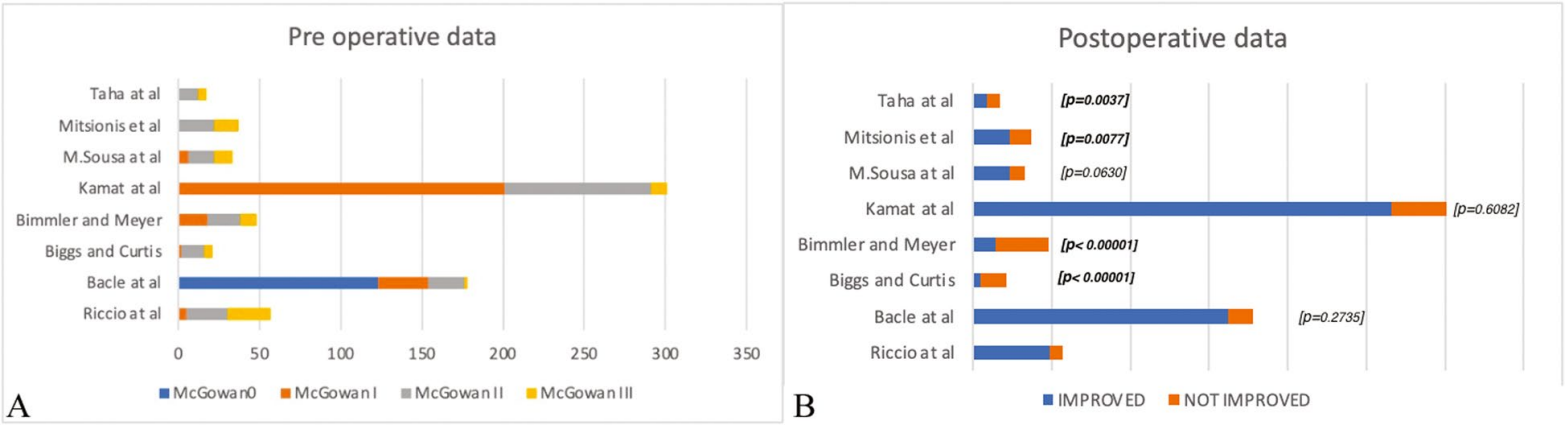
**A** Comparison between preoperative assessment of Riccio et al. data and other studies considered. **B** Comparison between postoperative assessment of Riccio et al. data and other studies considered. *P* value is calculated with chi square test

**Fig. 6:**
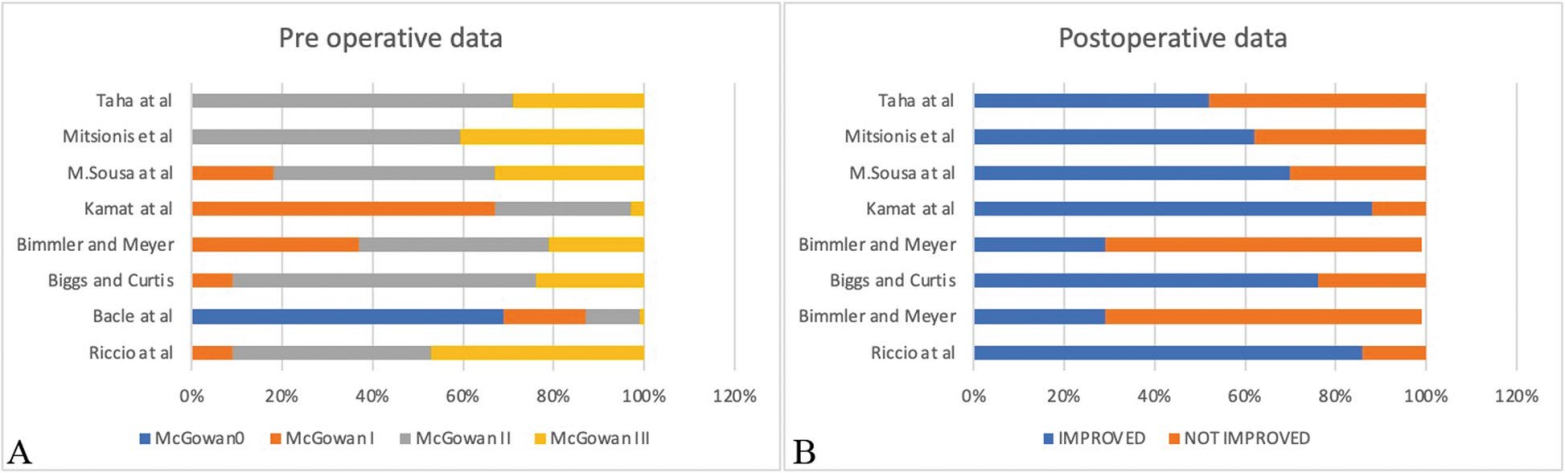
**A** Preoperative and (**B**) postoperative assessment in percentage of the studies

Among the 66 patients, nine were lost to follow-up. A total of 57 patients with a mean age of 58 (range 42–86) were included in the study, with a minimum follow up of 12 months. Table 1 shows the population characteristics: 37 males (64.9%), 20 females (35.1%), 54 mono-lateral cases (94.7%), 3 bilateral cases (5.3%), 35 dominant hand cases (61.4%), 19 non-dominant hand cases (33.3%), 3 bilateral cases (5.3%). Patients were evaluated after surgery and for at least 12 months, data reported an average McGowan’s classification score of McGowan I (8.8%), McGowan II (43.8%), McGowan III (47.4%); the Messina’s criteria determined “fair” (8.7%), “good” (43.8%), “excellent” (47.4%). At 6-month follow up, we analyzed the recovery of sensibility in compliance with the British medical research council S2 (1.7%), S3 (12.28%), S3 + (56.4%), S4 (29.82%) (Table 7), postoperative results according to initial stage are displayed in Table 8. All patients were able to resume their daily activities and professions. The analysis (Table 9) showed a significant difference in preoperative development with Bacle et al. (*p* < 0.00001); Kamat et al. (*p* = 0.002805); insignificant preoperative development for Biggs and Curtis(*p* = 0.286347); Sousa et al. (*p* = 0.450257); Mitsionis et al. (*p* = 0.450257); Taha et al. (*p* = 0.327513); Bimmler and Meyer(0.002805); a remarkable post-operative difference was determined for Biggs and Curtis (*p* < 0.00001); Mitsionis et al. (*p* = 0.0077); Taha et al. (*p* = 0.0037); Bimmler and Meyer (< 0.00001); non significant postoperative differences for Bacle et al. (*p* = 0.2735); Kamat et al. (*p* = 0.6082); Sousa et al. (*p* = 0.0630) (Fig. 5A,B). A comparison in percentage of the studies is exhibited in Fig. 6.

**Table 7:** Principal results from our database

McGOWAN'S		MESSINA'S		POSTOPERATIVE HEMATOMA		BMRC		RECURRENCE		Sensitive Scar	
1	8.8%	FAIR	8.7%	YES	5.3%	S2	1.7%	NO	100%	YES	10%
2	43.8%	GOOD	43.8%	NO	94.7%	S3	12.28%	YES	0%	NO	90%
3	47.4%	EXCELLENT	47.4%			S3 +	56.14%				
						S4	29.82%

**Table 8:** Comparison of the postoperative results according to preoperative

	PREOPERATIVE GRADE						
Mc GOWAN	I		II		III		p value
	n	%	n	%	n	%	0.140032
EXCELLENT	2	3.50%	17	29.80%	8	14%	
GOOD	3	5.30%	7	12.30%	12	21%	
FAIR	0	0%	1	1.80%	7	12.30%	
POOR	0	0%	0	0%	0	0%

**Table 9:** Pre operative and post operative data of the different studies, Confronted with chi square test

PRE OPERATIVE DATA				POSTOPERATIVE DATA			
	Riccio et al	Bacle et al	p		Riccio et al	Bacle et al	p
McGowan0	0	123	**< 0.00001**	IMPROVED	49	162	0.2735
McGowan I	5	31		NOT IMPROVED	8	16	
McGowan II	25	22		tot	57	178	
McGowan III	27	2					
tot	57	178					
	Riccio et al	Biggs and Curtis			Riccio et al	Biggs and Curtis	
			p				p
McGowan0	0	0	0.286347	IMPROVED	49	5	**< 0.00001**
McGowan I	5	2		NOT IMPROVED	8	16	
McGowan II	25	14		tot	57	21	
McGowan III	27	5					
tot	57	21					
	Riccio et al	Bimmler and Meyer	p		Riccio et al	Bimmler and Meyer	p
McGowan0	0	0	0.002805	IMPROVED	49	14	**< 0.00001**
McGowan I	5	18		NOT IMPROVED	8	34	
McGowan II	25	20		tot	57	48	
McGowan III	27	10					
tot	57	48					
	Riccio et al	Kamat et al	p		Riccio et al	Kamat et al	p
McGowan0	0	0	**< 0.00001**	IMPROVED	49	266	0.6082
McGowan I	5	201		NOT IMPROVED	8	35	
McGowan II	25	90		tot	57	301	
McGowan III	27	10					
tot	57	301					
	Riccio et al	M.Sousa et al	p		Riccio et al	M.Sousa et al	p
McGowan0	0	0	0.450257				0.0630
McGowan I	5	6		IMPROVED	49	23	
McGowan II	25	16		NOT IMPROVED	8	10	
McGowan III	27	11		tot	57	33	
tot	57	33					
	Riccio et al	Mitsionis et al	p		Riccio et al	Mitsionis et al	p
McGowan0	0	0	0.450257				**0.0077**
McGowan I	5	0		IMPROVED	49	23	
McGowan II	25	22		NOT IMPROVED	8	14	
McGowan III	27	15		tot	57	37	
tot	57	37					
	Riccio et al	Taha et al	p		Riccio et al	Taha et al	p
McGowan0	0	0	0.327513				**0.0037**
McGowan I	5	0		IMPROVED	49	9	
McGowan II	25	12		NOT IMPROVED	8	8	
McGowan III	27	5		tot	57	17	
tot	57	17

### Complications, aggravations, recurrences

The techniques led to complications in three cases, 5.3% of patients presented postoperative hematoma that did not require surgery, nine cases presented with scar sensitivity at six months with spontaneous resolution at one year following adequate scar treatment, no cases of recurrence were reported.

## Discussion

Ulnar nerve entrapment at the elbow is the second most common cause of nerve entrapment in the upper limb [1, 4] with an incidence of 20.9 per 100,000 per year [29]. Surgical treatment is required in case of inefficiency of conservative treatment regarding clinical manifestations of the cubitus. Surgical techniques include simple decompression, decompression with anterior transposition (submuscular, intramuscular, or subcutaneous) medial epicondilectomy [13], endoscopic decompression [30]. All techniques have offered satisfactory outcomes [17]. In situ decompression is a less invasive surgical procedure, minor devascularization of the nerve [31–34], rapid recovery time [31] and preservation of the nerve’s anatomical position. It is possible to perform the procedure using either open in situ decompression (OISD) or endoscopic in situ decompression (EISD). Anterior transposition is indicated in presence of bony spurs, synovial swelling or nerve subluxation [35], albeit disadvantage of mayor nerve de-vascularization [17, 32, 33] and risk of injury to the medial antebrachial cutaneous nerve [36].It can be performed without or with the adipo fascial flap. Medial epicondylectomy may yield post-operative tenderness, pain, weakness of the pronator and flexor. Numerous studies have been performed to assess an optimal technique but to date, no general consensus has been reached [17]. In 2014 a meta-analysis comparing the four main techniques (OISD-EISD-Subcutaneous anterior transposition- Submuscular anterior transposition) conducted by G.Bacle et al. showed that regardless of the choice of techniques adopted, effective treatment for the cubital syndrome has been demonstrated, with a satisfactory outcome of 85–95% [22]. In 2017, Ayesha Yahya et al. [36] conducted a study involving numerous members of the American Society for Surgery of the Hand (ASSH), who were required to answer questions regarding different scenarios of cubital syndrome presentation, to better understand surgical indications for treatment, concluding that, open in situ decompression was more favorable in the case of ulnar nerve subluxation [27]. Open versus endoscopic in situ procedures were analyzed in a meta-analysis (2019) by Vadim A. Byvaltsev and colleagues demonstrating no significant differences in the primary outcome, a relevant difference was observed regarding scar tenderness and elbow pain, which revealed a remarkably lower incidence in EISD [37]. In 2021, a review for anterior ante-position by E.Ergen et al. reported 89% of patients expressing satisfaction following treatment in an 11.5- year follow up involving 82 patients [38]. Choudhry IK et al. showed that both tension in the ulnar nerve and the pressure around the nerve decreased with transposition while in-situ decompression surgery revealed decreased pressure but remaining tension [39, 40]. Major cause of recurrent symptoms after anterior subcutaneous is perineural scarring. Use of a vascularized adipose flap to secure the anteriorly transposed ulnar nerve can help reduce nerve adherence and may enhance nerve recovery. In the present study, we retrospectively reviewed the long-term outcomes of ulnar nerve anterior subcutaneous transposition secured with an adipose flap (57 patients). We consider this technique as the evolution of subcutaneous transposition with a considerably low recurrence rate (0% in our database) and with a result calculated with Messina’s score as “good” or “excellent” in 91.2% of patients. Regarding comparison of previous literature, our data showed statistical differences in postoperative outcomes in relation to some studies (Biggs and Curtis [23]; Bimmler and Meyer [24], Mitsionis GI, Manoudis GN et al. [25]; Yahya A, Malarkey AR et al. [27]), due to a more effective surgical technique such as transposition plus adipofascial flap coverage. Moreover, we found no significant outcomes regarding postoperative developments compared to other investigations (Bacle G, Marteau E, Freslon M et al. [22]; Kamat AS, Jay SM, Benoiton LA et al. [28], Sousa M, Aido Rat et al. [26]), owing to a higher number of treated low grade McGowan patients, in contrast with more severe grade McGowan patients treated in our Department. Such data are confirmed by the significant differences in preoperative assessment, considering the same studies with an evident difference in McGowan grade enrolment (Kamat and Bacle: *p* =  < 0.00001) and M.Sousa: *p* = 0.0630.. The use of a scar tissue barrier, such as adipofascial vascularized flap, during ulnar nerve transposition reduces the incidence of scar and produces better outcomes [41]. Many authors [42, 43] have demonstrated the regenerative effects of adipose tissue flaps on peripheral nerves following crush injuries, and Strickland and colleagues [44] retrospectively examined the effects of hypothenar fat flaps on recurrent carpal tunnel syndrome, showing excellent results regarding this procedure. It is hypothesized that adipose tissue provides not only adipose-derived stem cells but also a rich vascular bed on which nerves will regenerate, via paracrine effects and the secretion of a range of neurotrophic factors such as the nerve growth factor (NGT), insulin-like growth factor 1 (IGF-1), neurotrophin-3 (NT-3) and -4 (NT-4), and basic fibroblast growth factor (bFGF). Increased neurotrophic factor expression results in an increase in axon sprouting, improves regeneration of the nerve and decreases inflammatory infiltrates. Moreover, adipose tissue may increase the production of neurotrophic factors (BDNF, GDNF) by the host Schwann cells [45]. This study was limited by its retrospective nature, which reduced access to preoperative objective and subjective data. Moreover, the large sample size demonstrated the advantageous effects of an adipofascial flap in preventing postoperative perineural scarring.

## Conclusion

In conclusion, the vascularized adipofascial flap is a viable option for securing the anteriorly transposed ulnar nerve. Outcomes in this study demonstrated the efficacy of this technique as symptoms resolved or improved, and most patients reported satisfaction with long-term surgical outcomes. The adipofascial flap may have additional advantages, such as a pliable, vascular fat envelope mimicking the natural fatty environment of peripheral nerves and creating desirable micro-environmental conditions to contribute to neural regeneration via axon outgrowth. The data suggest the applicable technique as a valuable option for surgical management of severe cubital syndrome.

## Data Availability

The datasets used and/or analysed during the current study available from the corresponding author on reasonable request.
